# Utilizing single-cell RNA sequencing for analyzing the characteristics of PBMC in patients with Kawasaki disease

**DOI:** 10.1186/s12887-021-02754-5

**Published:** 2021-06-14

**Authors:** Xue Fan, Yuhan Zhou, Xin Guo, Mingguo Xu

**Affiliations:** 1 The Department of Pediatric Cardiology, Shenzhen Children’s Hospital of China Medical University, Shenzhen, 518038 China; 2 Department of Pediatric, The Fifth Affiliated Hospital (Zhuhai) of Zunyi Medical University, 519100 Zhuhai, China; 3 The Department of Pediatric, Shenzhen Children’s Hospital of China Medical University, Longgang District Maternal and Children Health Care Hospital, Shenzhen, 518038 China

**Keywords:** Kawasaki disease, Single-cell RNA sequencing, PBMC, Immune cell

## Abstract

**Background:**

Kawasaki disease (KD) is the main cause of acquired heart disease in children and can lead to coronary artery lesions. This present study was designed to analyze the characteristics of KD peripheral blood mononuclear cells (PBMC) through single-cell RNA sequencing (scRNA-seq) and to explore the potential molecular mechanism of KD.

**Methods:**

PBMC was collected from one healthy child and one KD patient, and was used to single-cell RNA sequencing for cell clusters identification and differently expressed gene (DEG) determination. GO function enrichment analysis of DEG in B cell and T cells were performed to explore the most active biological function in KD immune cells.

**Results:**

Twelve cell clusters can be identified in two samples. Compared with healthy child, naive CD8+ T cell, T helper cell and B cell in KD child were decreased, mainly immune-related T cells, and natural killer T (NKT) cell were increased. Cell activation, lymphocyte activation and regulation of immune system process were 3 GO function shared by all four types of T cells and B cell.

**Conclusions:**

Immune cell disorder appears in the KD patient at single cell level by scRNA-seq.

**Supplementary Information:**

The online version contains supplementary material available at 10.1186/s12887-021-02754-5.

## Background

Kawasaki disease (KD), also known as cutaneous mucous lymph node syndrome, is an autoimmune disease with systemic diffuse vasculitis as the main symptom, which tends to occur in children under 5 years old [1]. As the most common cause of acquired heart disease in developed countries, KD is prone to cause coronary artery damage, including coronary dilatation and coronary aneurysms, and may even lead to myocardial ischemia, myocardial infarction and sudden death [2]. In recent years, many studies have suggested that the pathogenesis of KD is significantly correlated with infection, genetic susceptibility and immune response, and there are on-going studies focusing on its treatment based on different pathogenesis hypothesis. According to previous report, the acute phase of KD occurred with severe immune dysfunction, the activation of the immune system and inflammation factor of the cascade amplification effect is considered to be the main characteristics of KD. A certain number of researchers believe that KD is caused by pathogenic bacteria infection, leading to abnormal activation of the immune system and causing cascade release of inflammatory factors [3]. Recent studies have shown that the regulatory T cell is an important marker in determining the severity and susceptibility of KD [4]. In addition, lymphocyte subsets and immunoglobulin level are existed laboratory markers to differentiate KD from other febrile infectious diseases and healthy children [3].

Advances in next-generation sequencing technologies have recently made it possible to study the immune system at single cell level. Tang [5] et al. first published single cell transcriptome sequencing (scRNA-seq) technology in 2009, which has enabled high resolution mapping of cellular heterogeneity, development, and activation states in diverse systems. scRNA-seq is to amplify the trace transcriptome RNA of isolated single cells for high-throughput sequencing to obtain the expression profile of the complete transcriptome in the single cell level, thus revealing the molecular regulatory mechanisms of specific biological processes and disease processes [6, 7]. This technique is of great significance for the discovery of new therapeutic targets for cardiovascular diseases. At present, there are few reports on scRNA-seq of peripheral blood mononuclear cells (PBMC) in KD.

In this study, scRNA-seq was applied to PBMC of a KD patient and a healthy control, and bioinformatics analysis were performed to explore the cell cluster differences and DEGs that may affect the development of KD, so as to provide new targets for KD treatment.

## Methods

### Enrollment of participants and collection of samples

All manipulations were approved by the ethics committee (201908803) at Shenzhen Children’s hospital and written informed consent was acquired from the guardians of all donors. A total of 2 subjects, including 1 normal child and 1 KD patient, were recruited from Shenzhen Children’s Hospital. The child in the observation group is 1 year old girl from the department of Cardiovascular Section, whose main symptoms are continuous fever for 5 days and rashes for 3 days. The patient is conformed to the 2017 American Heart Association (AHA) KD diagnostic Guidelines and was not treated with any drugs prior to hospitalization [1]. The sample serum was collected on the day the patient was hospitalized before IVIG therapy. In addition, this patient does not occur CALs and is not IVIG resistance. The normal child was collected randomly in the child Health Section at the same stage, who is a 5 years old boy. The whole blood PBMC suspension was prepared by taking 3-5 ml peripheral blood of the children into the EDTA anticoagulant tube, and then we further prepared the gel bead in emulsions.

### Transcriptome amplification, library construction and sequencing

The preparation and cell suspension of PBMC were performed referred to the method section by professor Noa Bossel Ben-Moshe [8]. The mRNA of the cell forms cDNA under the action of reverse transcriptase, the cDNA synthesis and library construction were performed based on the10× Genomics Single-Cell 3′ Library V2 Kit. Then, these two cDNA libraries were sequenced as 100 bp paired-end reads on a BGISEQ-500 sequencer. Following, CellRanger v2.0 software was performed to process the raw FASTQ files, align the sequencing reads to the GRCh38 reference transcriptome build via STAR and generate a filtered UMI expression profile for each cell [9].

Data from two samples were merged into one gene cell barcode matrix. The number of genes, UMI counts and percentage of mitochondrial genes were examined to identify outliers. As an unusually high number of genes can result from a ‘doublet’ event, in which two different cell types are captured together with the same barcoded bead, cells with > 90% of the maximum genes or < 200 were discarded. Cells containing > 7.5% mitochondrial genes were presumed to be of poor quality and were also discarded.

### Cell clustering

The single-cell data analysis was performed by the Seurat v2.0.1 package [10], after quality control and filtering. Highly variable genes were calculated with the Find Variable Genes method of the Seurat package and 2000 genes were used for further analysis including principal components analysis (PCA).

The principal components were used for cluster identification using uniform manifold approximation and projection (UMAP) algorithm. For each clusters, the marker genes were identified using the FindConservedMarkers function as implemented in the Seurat package (logfc.threshold > 0.25 and minPct> 0.25). Then, clusters were remarked to a known cell type according to Cell Marker database [11].

### Gene different expression and function enrichment

Differently expressed gene (DEG) across different samples or cluster were identified using the FindConservedMarkers function in Seurat by parameters of ‘logfc. Threshold > 0.5, minPct> 0.25 and Padj≤0.05. GO and KEGG pathway analysis [12] were performed using Gene Set Enrichment Analysis (GSEA) with FDR < =0.05 as considered to be significant enrichment [13]. Function Circus plot was drawn by R version 4.0.3.

## Results

### Clinical symptoms and laboratory data

The main clinical symptoms of the KD patient are fever, rash, bilateral nonexudative conjunctives, erythema of the lips and oral mucosa and enlarged lymph node in the right neck. In the blood test, we can observe the similar pattern that neutrophile granulocyte count and percentage were also higher in the KD child than normal child far beyond the normal range (Table 1). Besides, Platelets count and C-reactive protein, which may be referred to as thrombocytosis and inflammation marker in body, were also beyond normal range. The data of ESR and PCT were all significantly increased on the fifth day during the course of disease. In addition, no significant changes were observed in biochemical routine.

**Table 1:** Blood result of KD patient and normal control

	Normal	KD
Leukocyte counts (10^9/L, 5–12)	8.74	22.59
Neutrophile granulocyte counts (10^9/L, 2–7)	4.65	16.36
Lymphocytes counts (10^9/L, 0.8–4)	3.22	4.81
Monocyte counts (10^9/L, 0.12–1)	0.26	0.75
Eosinophil granulocyte counts (10^9/L, 0.02–0.5)	0.61	0.64
Basophil granulocyte counts (10^9/L, 0–0.1)	< 0.01	0.03
Lymphocytes (%, 20–40)	36.8	21.3
Monocyte (%, 3–10)	3	3.3
Eosinophil granulocyte (%, 0.5–5)	6.9	2.8
Basophil granulocyte (%, 0–1)	0.1	0.1
Neutrophile granulocyte (%, 50–70)	53.2	72.5
Red blood cell (10^12/L, 3.5–5.5)	4.56	3.8
Hemoglobin (g/L, 110–160)	1.28	97
Platelets counts (10^9/L, 100–300)	253	391
C-reactive protein (mg/L, 0–10.0)	not available	48

### A single-cell transcription atlas of PBMC in KD child

**Fig. 1:**
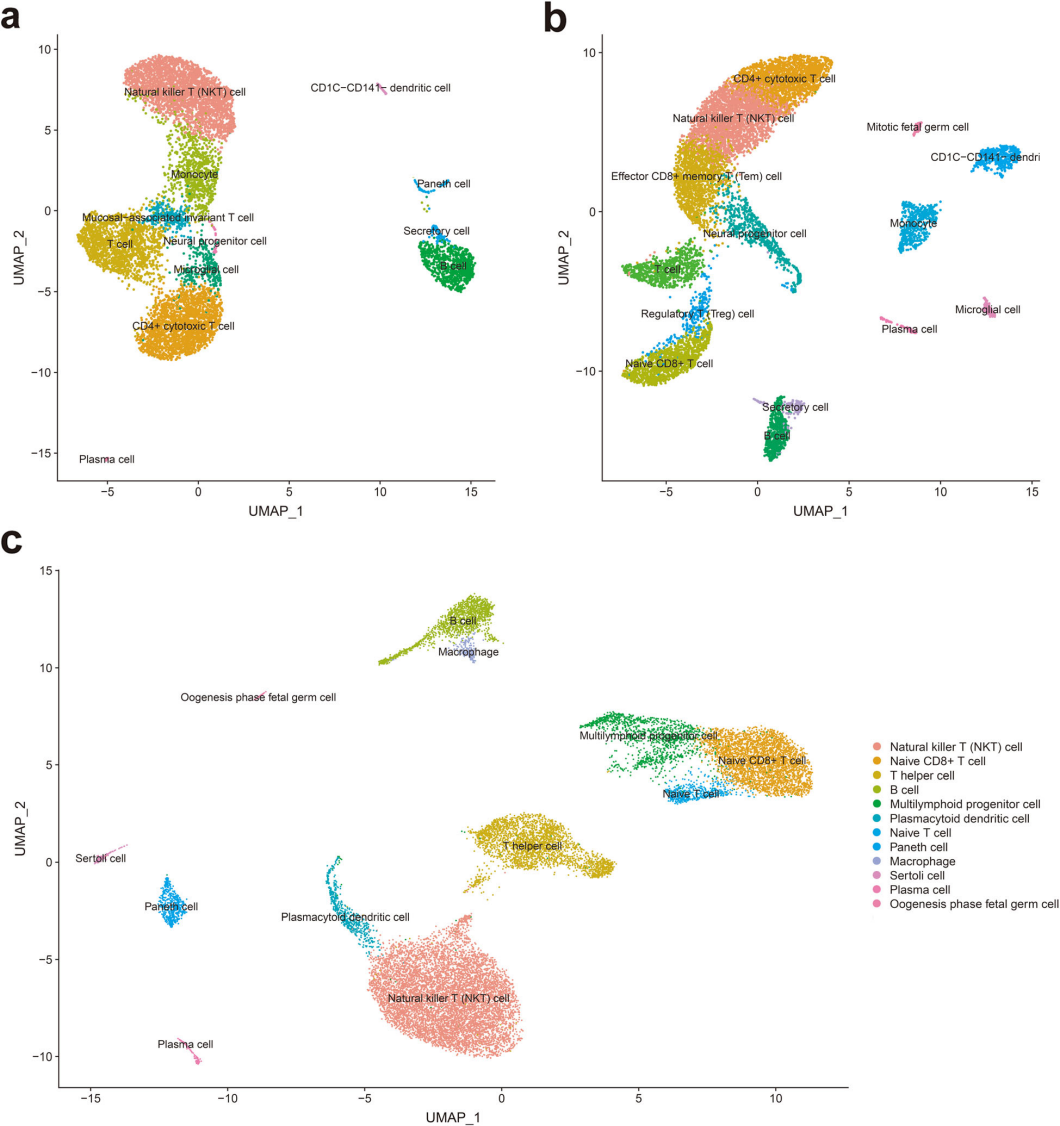
Cell types identified in peripheral blood mononuclear cells (PBMC) by uniform manifold approximation and projection (UMAP). **a** 12 cell clusters in normal child PBMC sample (*n* = 8941). **b** 14 cell clusters in KD child PBMC sample (*n* = 10,256). **c** 12 cell clusters in two samples (*n* = 19,197), clustered into populations as indicated

After stringent quality control and filtering by multiple criteria, transcriptomes of 10,256 and 8941 single cells from the KD child and normal samples were acquired, with a mean of 22,585 and 20,266 genes per cell detected, respectively (Supplemental Table S1). Based on principal component analysis (PCA), UMAP algorithm was used to cluster cells with similar expression patterns by dimension reduction. In this study, the two samples were classified into 12 and 14 clusters (Fig. 1 a &b), respectively. For the healthy child, the dominant cell clusters were natural killer T (NKT) cell, CD4+ cytotoxic T cell, T cell, monocyte and B cell. But in the KD child, effector CD8+ memory T (Tem) cell and naive CD8+ T cell replaced monocyte and B cell as the top 5 dominant cell clusters. Then, two single-cell datasets were merged to enable a systematic comparison between KD and normal child. We found no specified unique cell cluster in either sample and based on the expression gene markers (Marker genes refer to CellMarker Database), 12 cell clusters can be identified in Fig. 1c. NKT cell, naive CD8+ T cell, T helper cell, B cell and multilymphoid progenitor cell are the most **relative** abundance cell type.

### Differences in immune cell subpopulation existed in the KD child

**Fig. 2:**
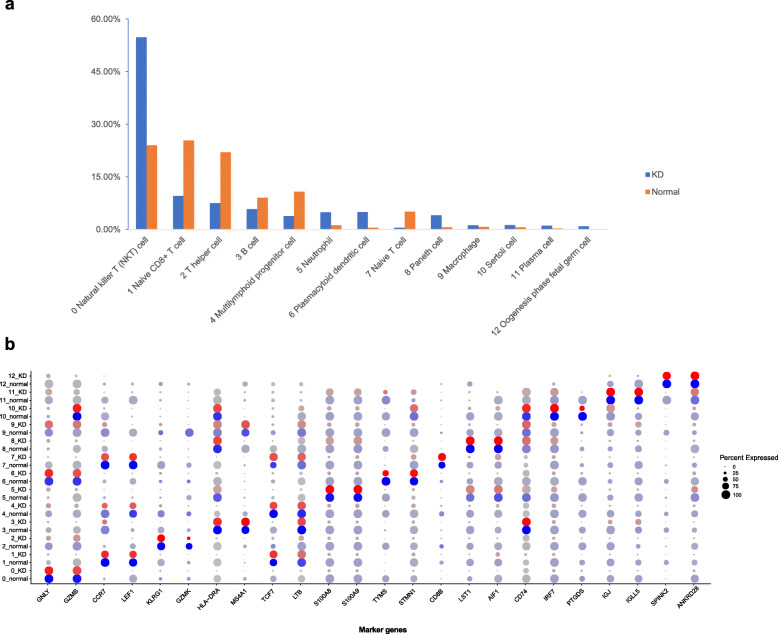
Bar graph of cell cluster different (**a**) and dot plot of top2 marker genes expression of cell clusters (**b**) in two PBMC samples. Dot size indicates proportion of cells in cluster expressing each marker gene, color indicates the relative expression level (low to high reflected as blue to red) based on UMI in each cell population. Top two marker genes are order as cluster 0 (*GNLY* & *GZMB*) to cluster 11 (*SPINK2* & *ANKRD28*). 0 Natural killer T (NKT) cell, 1 Naive CD8+ T cell, 2 T helper cell, 3 B cell, 4 Multilymphoid progenitor cell, 5 Plasmacytoid dendritic cell, 6 Naive T cell, 7 Paneth cell, 8 Macrophage, 9 Sertoli cell, 10 Plasma cell and 11 Oogenesis phase fetal germ cell

By comparing the composition of cell subpopulations between KD and healthy child, we found that immune-related cell clusters, example cluster 0 Natural killer T (NKT) cell and cluster 5 Plasmacytoid dendritic cell, were increased (Fig. 2a). Compared to the healthy child, these cell subpopulations (1 Naive CD8+ T cell, 2 T helper cell, 3 B cell, 4 Multilymphoid progenitor cell and 6 Naive T cell) in KD child PBMC were decreased, mainly immune-related T cells in Fig. 2a. On the whole, the above results indicate that differences in immune cell subpopulation existed in the KD child.

The two most abundant marker genes in each cluster are selected and displayed by dot plot in the two samples (Fig. 2b). There are only total 22 marker genes instead of 24, owing to some marker genes shared by 2 clusters. Marker genes *CCR7* and *MS4A1* were shared by cluster 1 and 6, cluster 3 and 8, respectively. We found that *IGJ* and *IGLL5* in plasma cell were significantly increased in the KD child compared to normal child with a 34 to 45 folds change, respectively (data not shown).

### KD disease states up-regulate gene expression and function in B and T cells

**Fig. 3:**
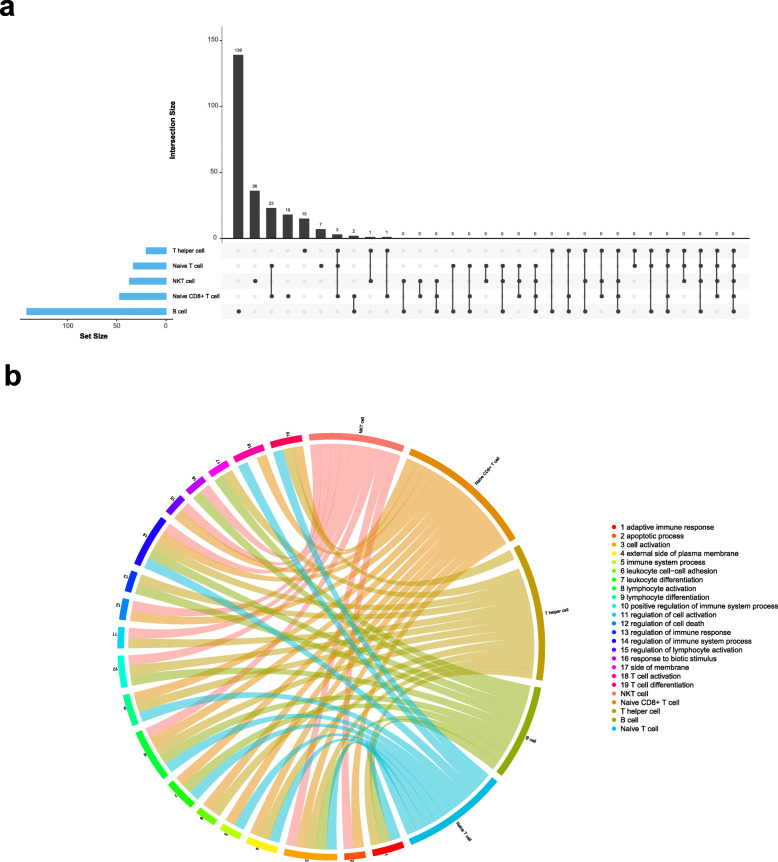
DEG and function in B cells and four T cells. **a** Upset of DEG in five cell types. X-axis represents DEGs in each cell type; Y-axis represents number of DEGs (FDR < 0.05). **b** Circus plot showing DEG set enrichment analysis using GO annotations reveal 19 term shared in at least 2 cell types. Significant GO term is declared at false discovery rate (FDR) < 0.05. The legend is the name of each GO term corresponding to each spoke of the circular plot

We defined gene with expression up-regulated in KD child compared with normal child as DEG. In order to explore the immune characteristics of children with KD, B cells and T cells were focused for detail analysis. As shown in Fig. 3a, DEG in T helper cell, naive T cell, NKT cell, naive CD8+ T cell and B cell increase gradually. By comparing co-upregulation of expressed genes, we found that three DEGs (*IL7R*, *CD3D* and *CD27*) were shared in naive CD8+ T cell, naive T cell and T helper cell, and only two DEGs (*CD69* and *LTB*), and single DEG (*GZMH*) and (*CD3G*) were common in B cell and naive CD8+ T cell, NKT cell and T helper cell, naive CD8+ T cell and T helper cell (Supplemental Table S2).

We further applied GO and KEGG enrichment analysis to explore the biological processes of the DEGs in B cell and four types of T cells. Enriched Function that shared by at least 2 types of T cells were displayed through circus plot (Fig. 3b). We found 3 KEGG pathways were only enriched in B cell subtype (Supplemental Table S3). Alternatively, 19 GO terms, which showed in circus plot, were classified into 17 biological process, 2 cellular components. There are 3 GO function shared by all four types of T cells and B cell as shown in Fig.3b: Cell activation, 8 Lymphocyte activation and 14 Regulation of immune system process.

## Discussion

KD is a common cause of vasculitis in childhood, which is characterized by fever and mucocutaneous features [14] . Current studies suggest that the regulation of T cell activation determines the susceptibility of KD and the severity of coronary artery lesions. However, despite emerging treatment options, the precise immune process of KD has remained unclear. Transcriptome is an important pathway linking the genome to the proteome and can provide new methods for the diagnosis, treatment and prognosis of diseases [15] . In previous study, a 13-transcript blood gene expression signature distinguished KD from other febrile conditions, including S100P, CD163 and RTN1, etc. [16]. And deep RNA sequencing was performed to reveal 1074 differentially expressed RNAs to provide direction for future etiology studies. At the transcriptomic level, we can further differentiate into more detailed subsets of cells based on different gene expression patterns [17–19]. scRNA-seq would differentiate the cell types in a complex population combination, promote the recognition of new cell types, and contribute to the understanding of the physiological processes of KD and the exploration of novel treatment options [20] .

Our study introduced a typical child PBMC example for single cell transcriptome study. We found that in healthy child, the dominant cell clusters are NKT cell, CD4+ cytotoxic T cell, T helper cell, CD8+ memory T cell and naive CD8+ T cell. These dominant cell clusters in healthy child were different from **healthy** adults, of which the dominant cell clusters were T cell, B cell, CD14+ monocyte, CD16+ monocyte and natural killer cell [21]. Back to the KD child, though no significant new cluster shown in the KD child compared to the healthy child, we found that the proportion of main cell clusters shifts in the KD child, with more NKT cells, plasmacytoid dendritic cell and less CD8+ T cells, T helper cells and B cells. We also observed that multilymphoid progenitor cells tends to decrease in KD child. Multilymphoid progenitor cells were believed to differentiate into multiple lymphoid cells including T cells and B cells. Multilymphoid progenitor cells were normally present in umbilical cord blood [21], and upper category progenitor cell was also found in PBMC (CellMarker Database).

Dendritic cell is the professional antigen presenting cell with the strongest function in the body. It can efficiently absorb, process and present antigens, and is the central link in initiating, regulating and maintaining the immune response. Cameron et al. [22] suggested that dendritic cell as antigen presenting cells are involved in KD arterial immune process. Miyabe et al. [23] demonstrate that Dectin-2–mediated induction of CCL2 production by macrophages resident in coronary arteries initiates vascular inflammation in a model of KD, suggesting the participation of innate immune system in initiating vasculitis. Another study suggests that mature arterial myeloid dendritic cell might be activating T cells and may be a significant factor in the pathogenesis of coronary arteritis in KD [24] . In addition, T cell regulation appears to be important at the tissue level for the resolution of inflammation in KD and evidence suggests that the Fc stimulates immature myeloid dendritic cell to expand the regulatory T cells [25] . With the study of dendritic cell at the molecular level, it has been found that it has great therapeutic potential in various autoimmune diseases, but the specific mechanism in KD need further exploration.

Furthermore, with the analysis of DEGs of immune cells in the KD and healthy control, we identified IL7R, CD3D and CD27 as the common DEGs that shared by three T cell clusters. IL7R encodes the receptor of interleukin 7, which is a crucial marker for T cell development and play essential role in immune competence. CD3D is associated with immune checkpoints [26] and may offer an possible new therapy for KD. CD27 is a marker of memory B cells and also detected on normal plasma cells, it is believed that CD 27+ memory B cells contributed to the pathogenesis of KD inflammation. In addition, we defined distinct subsets of NK T cells characterized by GNLY and GZMB. GNLY is found in cytotoxic granules in both CTL and NK cells, which is a member of the saposin-like protein family. It has been associated with a variety of infectious diseases and involved in the removal of virus such as varicella zoster and Epstein-Barr virus (EBV). One case report demonstrated that EBV infection could be considered as a suspected causative agent because of the potential effect on the immune system in KD [27]. GZMB represents a serine proteinase and plays a central role in killing human tumor cell lines, which can induce cell death, apoptosis [28]. A previous study found that GZMB gene silencing acts to inhibit MAPK signaling pathway through regulating the expressions of inflammatory factors, thus relieving the injury brought by Rheumatoid arthritis [29]. Moreover, studies have found that inhibiting MAPK signaling pathway activation can reduce the occurrence of the inflammatory response [30]. Collectively, we speculate that GNLY and GZMB may be potential targets for the treatment in KD.

After KEGG and GO annotation, we observed that the DEGs of T cells and B cells are specified enriched in Cell activation, Lymphocyte activation and positive regulation of immune system process. All the three shared biological process indicated that there existed abnormal activation of immune cells in molecular pathway level in the KD patient. Based on the pathogen theory of KD, the immune system is activated to eliminate the pathogen with stimulation of multiple T cells. When the pathogen replication is not well controlled, this continuous stimulation would lead to contraction of effector cells and cause their exhaustion [31]. CD27 is one of the marker for the immune tolerance pathway [32], the activation of CD27 and the enriched pathways indicated that immune checkpoint can be a possible new target for KD treatment.

Besides, MHC class II protein complex was significantly enriched only in B cell (Supplemental Table S3), and some studies had found that some MHC class II alleles had correlations with the probability of autoimmune diseases [33]. In addition, the DEGs of MHC class II has 10 genes in KD child B cells, including *HLA-DMA*, *HLA-DMB*, *HLA-DPA1*, *HLA-DPB1*, *HLA-DRA*, *HLA-DQA1*, *HLA-DQA2*, *HLA-DRB1*, *HLA-DRB5* and *HLA-DQB1* (Supplemental Table S2). The above results suggest that KD susceptibility is correlated with DM, DP, DQ and DR locus on HLA genes. Previous reports [34] have shown that HLA locus may play a potential role in the pathogenesis of disease in the process of antigen processing and presentation in MHC region, and it is believed that the expression of class HLA-I alleles is related to KD. These studies confirmed the HLA genes and MHC class II molecules are involved in the pathogenesis of KD, providing a new direction for the diagnosis and treatment of KD.

In summary, this study preliminarily explored the immune mechanism of KD by scRNA-seq technology, and explored relevant molecular markers and major enrichment function through bioinformatics analysis. These markers may be important targets for future treatment of KD. The sample size of this experiment is limited for solid conclusion, so it is necessary to further expand the sample size and proceed the study for further experimental research.

## Conclusion

In the present study, we identified immune cell difference in PBMC sample of KD and healthy child using scRNA-seq. Immune cell disorder appears in the KD patient at single cell level and blood test. The function of up-regulated genes expressed in B cell and four T cells in KD child is mainly cell activation, lymphocyte activation and regulation of immune system process. And this study is limited and further studies still need to confirm the functions of these DEGs in KD child.

## Supplementary Information

**Additional file 1: Table S1.** Statistical table of gene expression in single cell RNA-seq.

**Additional file 2: Table S2.** Different expression genes in B cell and T cell.

**Additional file 3: Table S3.** Function enrichment in B cell and four T cells.

## Data Availability

Single cell transcriptome data are in available at China National GeneBank Nucleotide Sequence Archive BioProject accession number CNP0001511.

## Abbreviations

KD: Kawasaki disease
scRNA-seq: Single-cell RNA sequencing
DEG: Differently expressed gene
PBMC: Peripheral blood mononuclear cells
